# Memories of John N. Brady: scientist, mentor and friend

**DOI:** 10.1186/1742-4690-6-48

**Published:** 2009-05-19

**Authors:** Cynthia A Pise-Masison, Susan J Marriott

**Affiliations:** 1 Laboratory of Cellular Oncology, Center for Cancer Research, National Cancer Institute, National Institute of Health, Bethesda, Maryland 20892, USA; 2 Department of Molecular Virology and Microbiology, Baylor College of Medicine, MS-385, One Baylor Plaza, Houston, Texas 77030, USA

## Abstract

Friends and colleagues remember John N. Brady, Ph.D., Chief of the Virus Tumor Biology Section of the Laboratory of Cellular Oncology, who died much too young at the age of 57 on April 27, 2009 of colon cancer. John grew up in Illinois and received his Ph.D. with Dr. Richard Consigli at Kansas State University studying the molecular structure of polyomavirus. In 1984 John came to the National Institutes of Health as a Staff Fellow in the laboratory of Dr. Norman Salzman, Laboratory of Biology of Viruses NIAID, where he was among the first to analyze SV40 transcription using in vitro transcription systems and to analyze regulatory sequences for SV40 late transcription. He then trained with Dr. George Khoury in the Laboratory of Molecular Virology NCI, where he identified SV40 T-antigen as a transcriptional activator protein. His research interests grew to focus on the human retroviruses: human T-cell lymphotropic virus type I (HTLV-I) and human immunodeficiency virus (HIV), analyzing how interactions between these viruses and the host cell influence viral gene regulation, viral pathogenesis and viral transformation. His research also impacted the fields of eukaryotic gene regulation and tumor suppressor proteins. John is survived by his wife, Laraine, and two sons, Matt and Kevin.

## Comments

**Figure 1:**
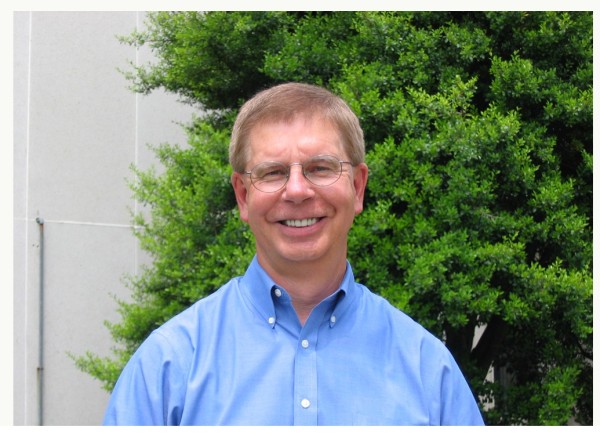
**John standing outside Building 41 on the NIH campus in 2006**.

**Figure 2:**
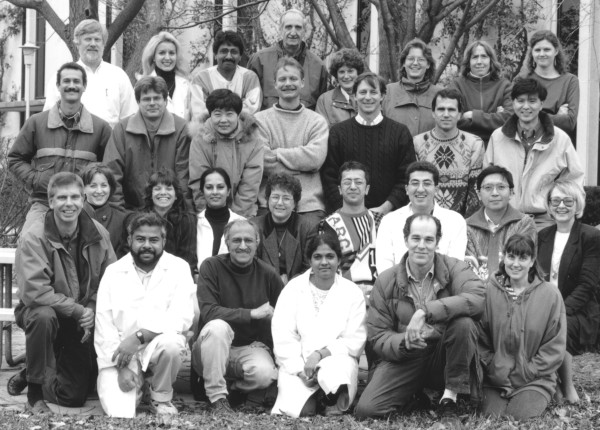
**People from the Laboratory of Molecular Virology assembled outside Building 41 on the NIH campus in February 1996**. John is kneeling in the lower left corner. Contributed by Gordon Hager (upper left corner).

John brought a formidable intellect and commitment to his longtime work at the NCI (Figure 1). His prolific career included over 200 research papers in very prestigious journals. He served on editorial boards for several virology journals, and was named to the International Retrovirology Association Advisory Board. John received numerous awards and recognition for his work including an NCI Intramural Award in 1988 for Innovative Research, and in 2002, he was named an NIH Senior Biological Research Scientist (SBRS) by the NIH Director, a highly competitive appointment reserved for researchers with outstanding achievements. In 1996, John was appointed adjunct Professor at George Washington University Institute for Biomedical Sciences. In addition to science, John had a love of baseball and served as a coach for and president of the Montgomery County Baseball Association (MCBA), an organization that promotes baseball for youth. These years of loving service will long be remembered by his sons, Matt and Kevin. John was dedicated to mentoring the many graduate students and post-doctoral fellows who passed through his lab, as well as young baseball players. His memory will long remain with his colleagues and with the researchers who were privileged to have him as a mentor (Figure 2). While these distinguished accomplishments are often used to measure professional success. John was so much more than that to many people who had the honor to work and train with him. To provide a glimpse into the man who impacted so many of us, some of the people who worked with him in various capacities over the years have provided their recollections of John here.

### Susan Marriott; Professor, Baylor College of Medicine

I visited the NIH in 1986 to interview for post-doc positions. I had done my PhD research in the lab of Dick Consigli at Kansas State Univ., the same lab where John received his PhD. John was famous in the Consigli lab for the meticulous work he had done on polyomavirus structural proteins. As graduate students we were often told to "look at the Brady papers" to see what a good experiment or good paper looked like. Although I had never met John, Dr. Consigli wanted me to visit him during my NIH trip and deliver greetings from the lab. When I stopped to see John I thought it would be a quick hello and nice to meet you. But we sat in his office for several hours talking about the Consigli lab and then about John's research interests. His energy and enthusiasm were infectious and as I flew back to Kansas I realized that I was more interested in his lab than those that I had gone to interview with.

**Figure 3:**
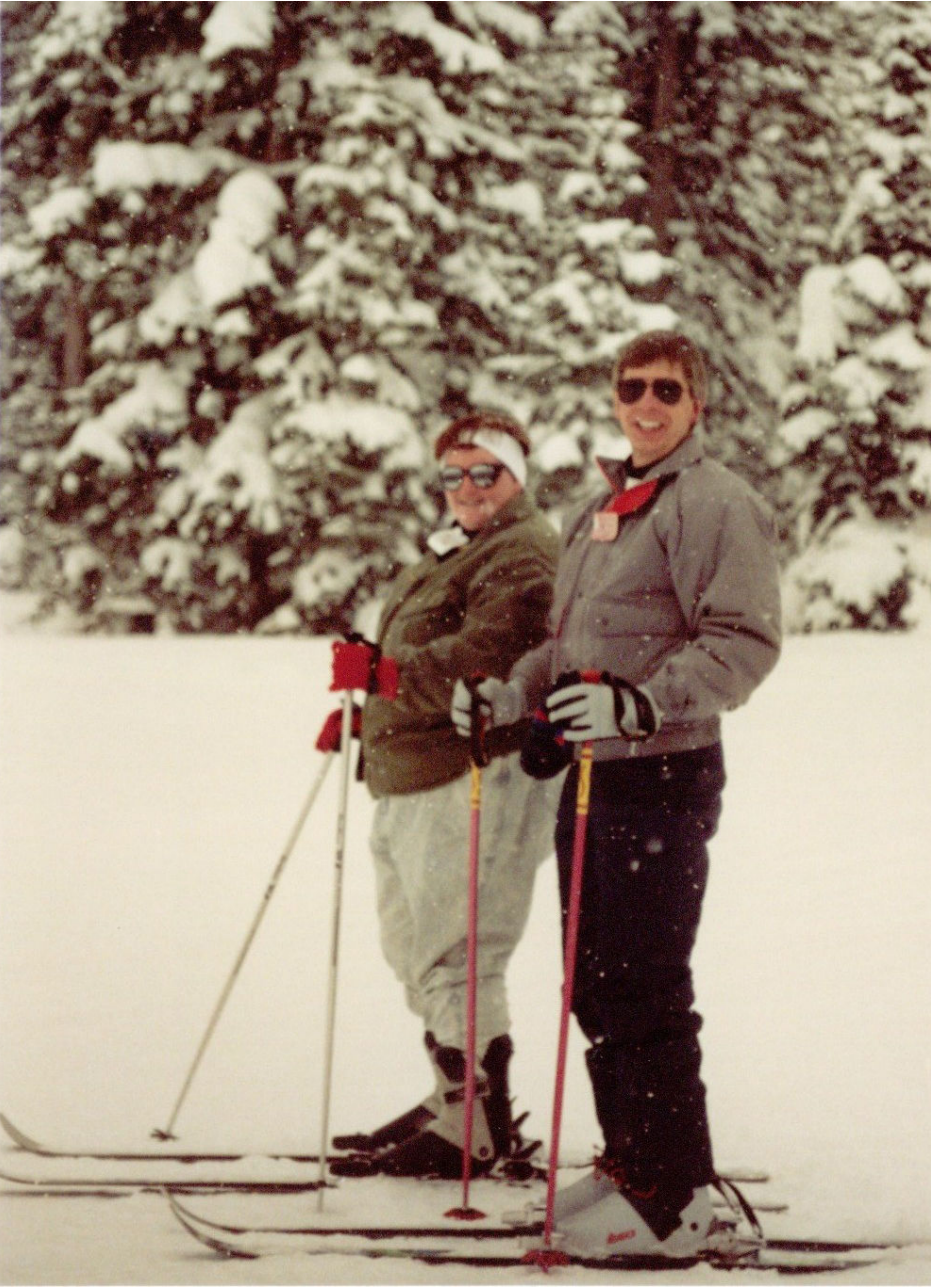
**Skiing at a Keystone Symposium meeting in Colorado, circa 1989**. Susan Marriott (left), John (right).

**Figure 4:**
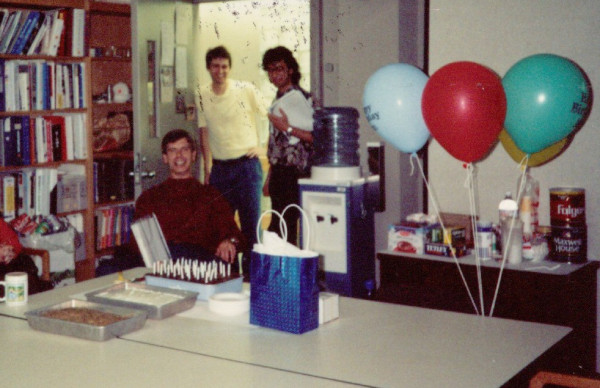
**John's 40^th ^birthday party in the infamous conference room where Friday morning lab meetings were held, Fatah Kashanchi (far right)**.

**Figure 5:**
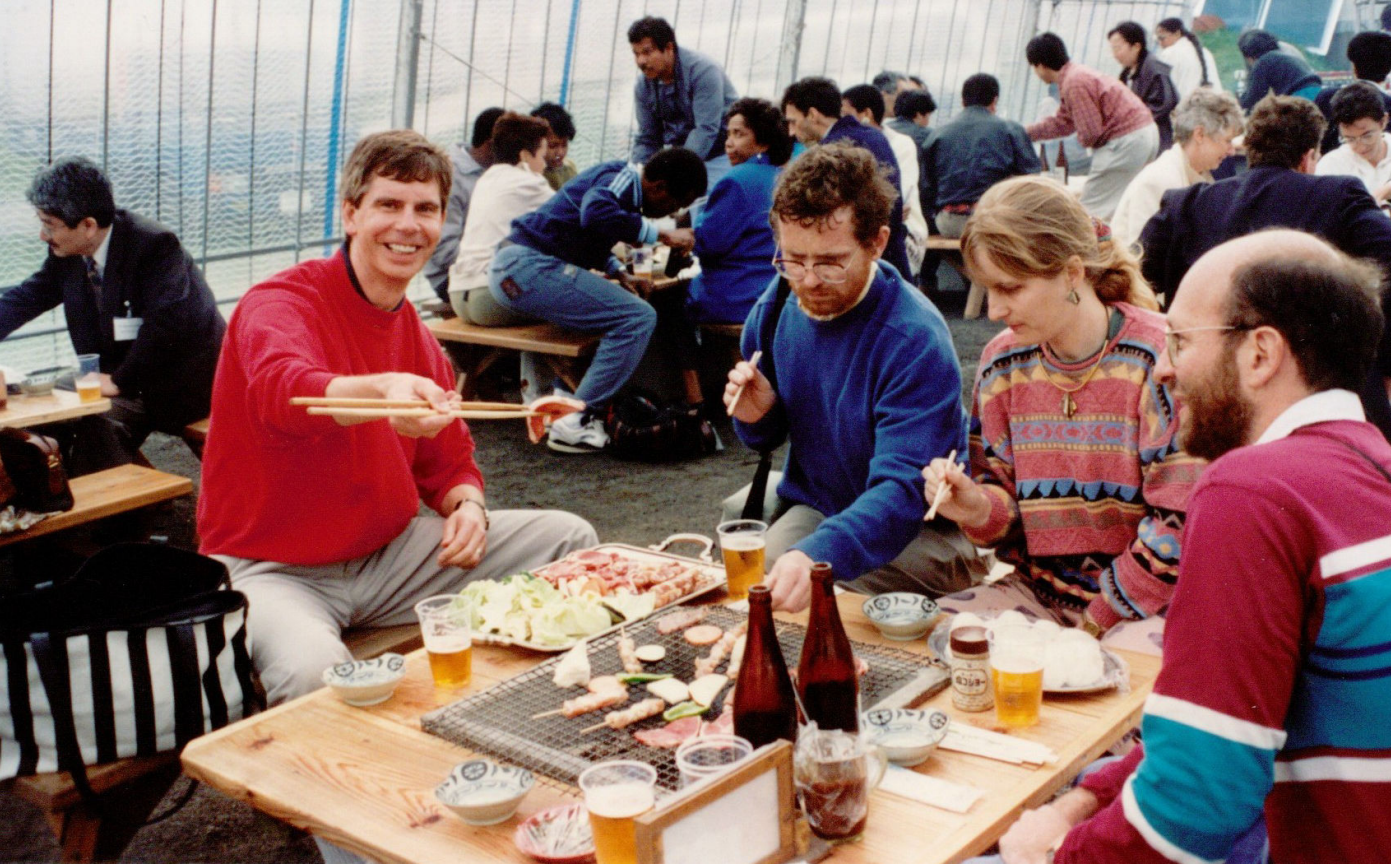
**John getting ready to cook his dinner at the HTLV meeting in Kumamoto, Japan (1992)**. Also at the table are Lee Ratner and Vincenzo Ciminale.

I ended up working with John for 4 years as a postdoctoral fellow. He taught me many things that a post-doc should learn and there were many good times (Figures 3, 4), but the most unique thing was that John gave me (and all of his post-docs, I think) an enormous amount of freedom to follow ideas that interested us. His only requirement was that we do good experiments. No one wanted to show up for lab meeting on Friday morning only to find out that their results were meaningless because they forgot a control. John promoted people in his lab by offering us opportunities to co-author review articles, represent the lab at meetings around the world (Figure 5), and he was very generous in letting us take research projects with which to begin our own independent careers.

During the 18 years since I left the Brady lab, John became a dear friend and colleague. I stopped by his lab to visit him along with his long-time colleague Mike Radonovich and to meet new lab members, whenever I was in Washington. I often thought it would be a quick stop to say hello, but it usually turned into a long visit with hours of talk about the latest experiments from both of our labs. The work from John's lab was always impeccable and I now tell my own students to "look at the Brady papers" to see what a good experiment looks like. John's unexpected death is a great loss to our field and a tragedy for his family. Personally, I will miss him dearly.

### Cynthia Masison; Staff Scientist, NIH/NCI

I was privileged to be a member of the "Brady Lab" for the past fourteen years, first as a postdoctoral fellow and then as a Staff Scientist. As a scientist I could certainly tell you about John's distinguished career and the significant contributions he has made to the fields of transcription and retrovirology. On a more personal level, I could tell you about the enthusiasm and energy John brought to discussions about science and his willingness to make time to discuss (and argue) about results and experiments. John's door was always open.

Others may rather hear about the fun and lighthearted conversations we had about John's other passion; baseball. John was always ready to argue about the Red Sox verses the Cardinals or give me advice about how to fix my son's batting stance or improve his pitching form. Better yet, I could tell you about John's memories of his good old days playing shortstop in school.

You might enjoy hearing the heated social and political debates that went on at lunch. John was an involved parent and community member. Concerned about the well being of his lab, his family and the community, he took the time to address issues at work, in the schools and in the greater community; true to his values and spirit.

I could talk about the fun and jokes that went on in the lab. Jokes played on John and by John. Or perhaps talk about the lab legends that will live on forever, like Scott's lab coat, John's attempt to make a yellow cake, or lab meetings in the "good old days". But voicing all the memories and telling all the stories still falls far short of conveying who John was and what he meant to so many of us. All who know him would agree that John was a genuinely nice guy and that it is such a tragedy that he died so young. John's science was a reflection of his life, rooted in humility, enthusiasm, generosity and integrity. He will be greatly missed.

### Peter Howley; Professor and Chair, Harvard Medical School

John had a wonderful sense of humor and was committed to people (trainees, colleagues, everyone). We will miss him for his science but more importantly for his friendship.

### Kuan-Teh Jeang; Senior Investigator, NIH/NIAID

**Figure 6:**
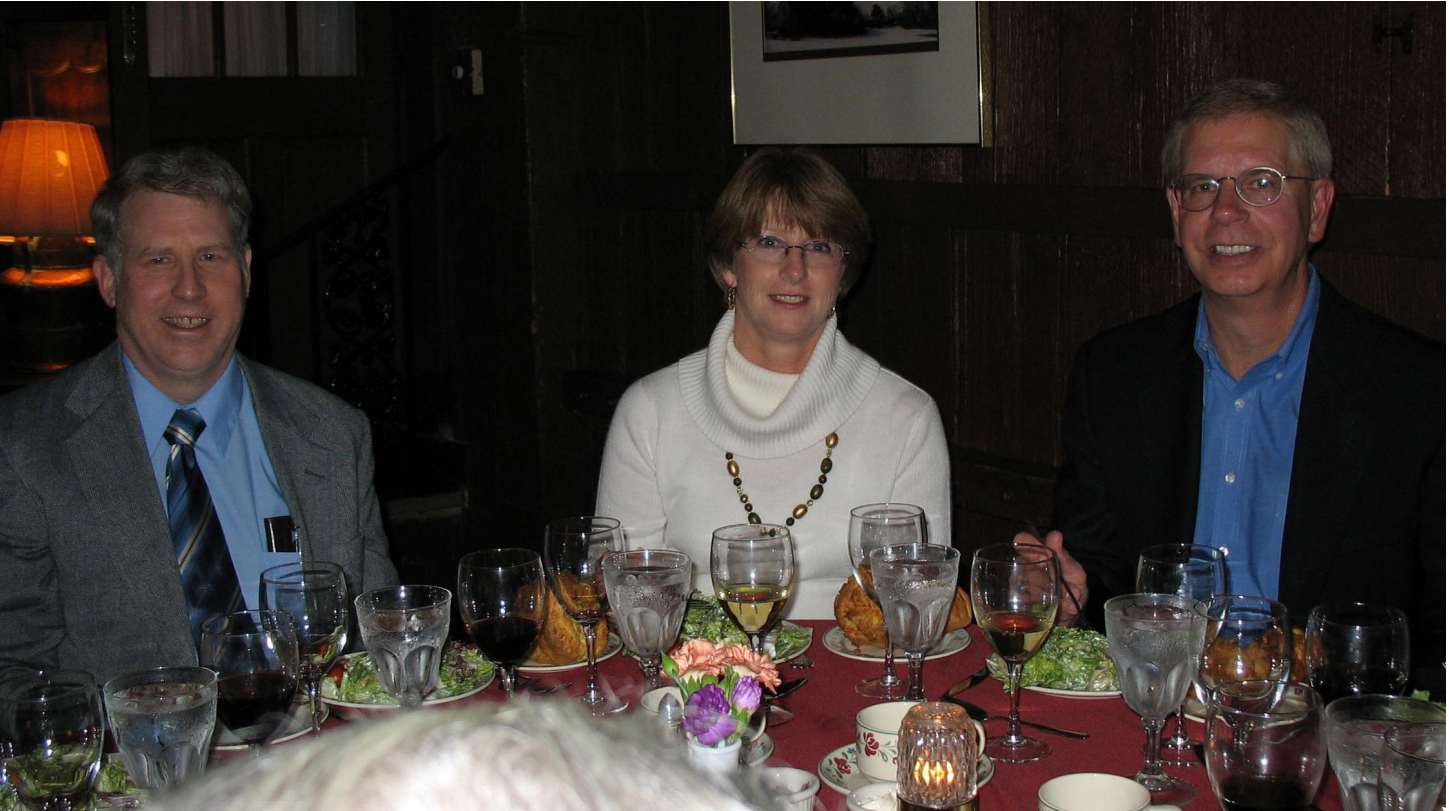
**John (right), his wife Laraine (center), and Carl Baker (left) at the dinner for the 2006 George Khoury lecture**. Contributed by Kuan-Teh Jeang.

After George Khoury passed on 22 years ago, John did most of the difficult work trying to hold the lab together. I admired how John tried to do right by everyone. After that, John and I ran the NIH George Khoury lecture together. It is always a great event, and I feel so gratified to have had the opportunity to work with John on the Khoury lecture (Figure 6). John was the quintessential "Mr. Nice Guy"; he was a great scientist; he was my friend, and he will be missed greatly.

### Kamel Khalili; Professor and Chair, Temple University

We lost a wonderful friend, and superb scientist. John's advice while I was in George Khoury's lab was key for my scientific achievement. I will miss him enormously.

### Michael Lairmore; Professor and Chair, The Ohio State University

I remember John as an outstanding scientist, but also as a devoted father and baseball coach. When we visited he never failed to mention his family and his love of baseball. He was a true Midwestern man with a compassionate view of the world.

### Scott Gitlin; Associate Professor, University of Michigan

John was a very good-hearted man who cared dearly about his family, his friends and his church. An important part of his life was spending time with his family and attending his son's sporting events. Despite the numerous invitations that he received, John preferred to stay "home" rather than spending a lot of time on the road giving extramural presentations.

John welcomed people to his laboratory and created an environment where everyone could contribute. John had high standards for the integrity and quality of the research that came out of his lab. He was committed to adding to the world's scientific knowledge by making sure that appropriate controls were included in all experiments, that results were reproducible and that relevant experimental conditions were used. He taught these values to all of his postdoctoral fellows, graduate students and technical staff, many of whom are pursuing successful academic careers across the United States. This all added to his reputation of being an honest scientist, a scientific leader in all of the viruses that he studied, and a genuinely nice guy.

In addition to his love of science and his family, I remember John's love for his Corvette. He picked me up from the airport in this car on the day that I came to interview with him-a very generous act in my opinion. John will be sorely missed by his family and friends, current and former laboratory colleagues, collaborators, and the scientific community as a whole. His legacy will be perpetuated by the accomplishments of his family and those scientists that participated in research with him.

### Renaud Maheiux; Professor, ENS Lyon

I spent almost three years in John's lab. He introduced me to the molecular biology field. I remember the hours that I spent in his office discussing my luciferase results. I had already performed the experiments 10 times but John always wanted me to repeat these experiments again and to change a little bit of this and a little bit of that. I remember how happy I was when John asked me to replace him at the Kagoshima HTLV meeting. That was his way of telling me that he trusted me, even if I was not a native English speaker, to present the lab's data. I remember how free I was, as a post-doc, to perform whatever I wanted and to collaborate with whomever I wanted. He provided me with all the reagents that were needed to succeed. I will always remember the last email he sent me two weeks ago, about an article we were writing together. He ended his mail by saying "what can I do for you"? This was John.

### O. John Semmes; Professor, East Virginia Medical School

John has played various mentoring roles for everyone he has had contact with. I will remember John as a quiet, unpretentious and an incredibly kind person. And these are far more important traits than his being the notable scientist that he was.

### Maureen Shuh; Associate Professor, Xavier University

While I never worked with John directly, my professional interactions with him felt very personal because of how he treated me. As a new postdoctoral fellow in David Derse's lab, I was rather intimidated by scientists in our field. Dave wanted me to call scientists for reagents and one of the first people I called was John. I have never heard a more friendly voice on the phone and John was so generous with reagents and assistance. I thought, "Wow, are all HTLV-I guys like him? I'm lucky I decided to enter this field." After Hurricane Katrina, John invited me to visit his lab at NIH and give a seminar. He thought it would be nice for me to think about science for awhile and get away from the Katrina mess. He was right on mark. To this day, I use John as the standard of I should behave and think as a scientist and as a person.

### Paul Lindholm; Associate Professor, Northwestern University

When I interviewed at the NIH, I met with several lab directors. I was intrigued by the virology projects in John's lab and he was very friendly and supportive. I chose to work in John's lab and found it to be a great place to learn science and work as a team. The high point of my week in John's lab was participating in the Friday morning lab meetings. John taught us by example how to think about our laboratory findings and how to take the next step. This was especially important for me, as I was previously trained in medicine and pathology and not directly in basic science. I fondly remember those Friday morning meetings and strive to have meetings like that in my own lab. He showed much enthusiasm and energy whenever I showed him my data, analyzed it with him, and planned the next experiments.

**Figure 7:**
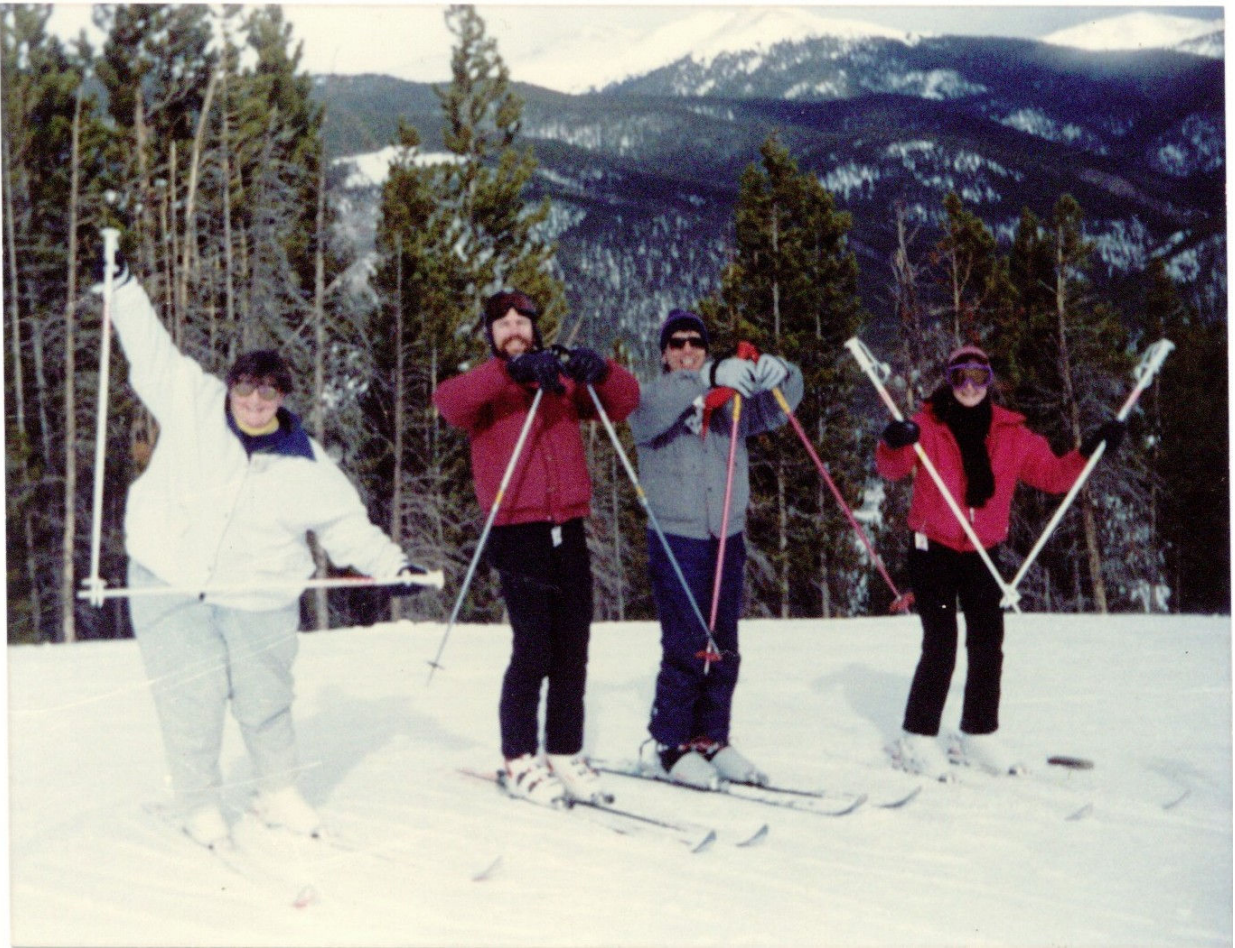
**Skiing at a Keystone Symposium meeting in Colorado (circa 1990) and spelling out the Laboratory of Molecular Virology (LMV) initials with ski poles**. Left to right: Susan Marriott, Paul Lindholm, John Brady, Deb Lindholm.

John was a great person and it was fun to be a member of his lab (Figure 7). He had a great sense of humor and enjoyed teasing the members of his lab. He used to tease me about going hiking in the Appalachian Mountains to "look at the leaves." That was his way suggesting that I could get back to work now. John encouraged me to work hard and make as much progress as possible. He used to tell me with a chuckle, "You can pay me now or you can pay me later." John taught me much through his enthusiasm and energy for science. When I heard of his passing, I realized that we had lost a great man and teacher in science.

### Cindy Morris; Associate Professor, Tulane University

When I came to NIH as a postdoctoral fellow in Dr. Flossie Wang-Staal's laboratory, Building 37 was being renovated and only limited laboratory space was available. I knew that I wanted to develop an in vitro transcription system to elucidate the mechanism by which Tat regulates HIV-1 transcriptional activation (pre-pTEFb era). Having a mission, I sought to find someone who might be able to teach me for a week or so until lab space became available. I found Dr. John Brady. I called him directly and arranged to meet him. When I came to his office, we talked for probably less than ten minutes and then he lead me to a bench and desk. We got started and I never left. When Flossie moved to California, John offered me an official spot in his lab and I eagerly accepted. I look back on those days with great fondness. I felt challenged and I loved the environment that John had created. John was always available to question, to comfort, to teach and to amuse. He was a kind and thoughtful leader and a good man. I remain forever grateful for how he influenced my life both personally and professionally. He would be pleased to know that I have not cried before any of my talks since I left his laboratory. "There's no crying in baseball," or in science. John taught me to persevere and remain optimistic against any odds. I will miss him and will keep him in my thoughts and prayers forever.

### Karl Munger, Associate Professor, Harvard Medical School

Even though I never worked with John directly, this is an enormous loss for the virus community. John was a wonderful guy, always with a smile and his Corvette in the parking lot. He was a man of integrity who was interested in research not the hoopla surrounding it. He showed that you can be a great scientist a really nice guy all at the same time!

### Chou-Zen Giam; Professor, Uniformed Services University of the Health Sciences

John and I joined the Laboratory of Molecular Virology at about the same time in the early 80's. The John I knew, in addition to being an excellent scientist, was a truly decent human being. John understood the doldrums and frustrations that could come from the daily practice of science and used humor to "spice" up the sometimes tedium of daily routines. I never heard John raise his voice or saw him lose his cool during the four years I was in LMV. In a lab full of strong personalities, that says a lot about John's character.

### David Derse; Senior Investigator, NIH/NCI

I am deeply saddened by this terrible loss. Although I knew John for over 20 years -first at scientific meetings, then often at NIH, and we held joint lab meetings for a short time recently – our conversations were usually pretty simple. We usually talked about work, the NIH news, and a few details about family and children were shared. With John, it wasn't all about John; it was almost like he thought there must be better things to talk about. When John went to meetings, it was to learn something new or to teach, not to promote himself. I went to his funeral on Saturday and similar things were said by the people who knew him outside of work – other baseball coaches, some of whom didn't know for many years that John was a well known research scientist. To them he was just a regular guy and he didn't talk about his work. To me John was the most generous, positive, and authentic person I have known. John leaves us with a great example, both as scientist and as an individual, that we can aspire to. I will miss him and I will remember his example.

### David Price; Professor, University of Iowa

I always looked forward to visiting with John when traveling to NIH for study section duty. He never failed to have an interesting finding that he was excited to share. These visits frequently ended with productive collaborations in which John was generous with authorship and credit. The HIV transcription community will miss his insightful contributions. He was one of the "good guys."

### Fatah Kashanchi; Professor, George Washington University

I worked with John for eight years in varying positions including, post doc and senior staff fellow. I felt a considerable amount of energy during those years. He essentially trained me to think scientifically, incorporate many controls into any given experiment, learn the biology of seemingly common proteins, such as Tat and Tax, and how they functioned in totally separate manners. During these eight years I worked with him on an almost daily basis, and those were some of the best times of my scientific life.

John played many roles in his lab, he was a mentor, a brother, at times a father figure, and always supported all his students, post docs, and staff. I could always go to him, under any circumstances, and he would respond positively, giving advice at many levels regarding science or everyday life. He therefore served not only as a scientific colleague, but also a close family member. He was a balanced person and he dedicated a considerable amount of time to science, to the people in his lab, as well as to his family and what his sons loved including baseball. It was very clear that anything he wanted to do had to be perfect, and he did it in a graceful and dignified manner.

John will be sorely missed among many of us who went through his lab and we intend to follow his footsteps and his long-reaching guidance. I know in my mind he will always be in my life, and when I'm in need of advice I will think, "What would John do."

### Kathleen Boris-Lawrie, Professor, The Ohio State University

John loved experiments. He loved controls, replicates, titrations, and interpretation of a solid data set. He held lofty standards for a dataset and gravitated toward challenging experimental approaches. He was passionate about the bench, and the pulpit less so. His loss is wrenchingly premature. From the calm of his very private world, I hope he knew he had the support of many.

### Sebastien Chevalier; Post-doctoral Fellow, NIH/NCI

John was a very nice man ... In the lab, if you passed him in the hallway, he would always ask with a big smile on his face, "how you doing, guy?"

When I discussed with him about results, he was very positive and he said: "yeah ...very interesting..." but 5 minutes later I would get an email to come to see him because he thought about something else ... of course other experiments to confirm for example that a monoclonal antibody recognized really the specific protein and not something else. I always had to think twice before presenting some results to him because he would find the weak point of that experiment very easily!!!

When I arrived in the lab, I proposed to buy an espresso machine with some other members of the lab. Of course, John contributed to that and the story about "how to make a good espresso "started. Each day, he would tell us that he didn't understand why but he didn't obtain the same coffee as us... strange because we were using the same machine, same coffee and same cup. So, finally, we had to have several training courses for how to make a good espresso and after 1 week of practice... He could make it!!! For sure, John could work at the NIH but not at Starbuck coffee!!!

